# The effect of electronic health records adoption on patient visit volume at an academic ophthalmology department

**DOI:** 10.1186/s12913-015-1255-8

**Published:** 2016-01-13

**Authors:** Jocelyn G. Lam, Bryan S. Lee, Philip P. Chen

**Affiliations:** Department of Ophthalmology, University of Washington, 325 Ninth Ave, Box 359608, Seattle, WA 98104 USA

**Keywords:** Ophthalmology, Electronic health record, Electronic medical record, Health information technology, Medical informatics, Health care delivery, Health law

## Abstract

**Background:**

Electronic health records (EHRs) have become a mandated part of delivering health care in the United States. The purpose of this study is to report patient volume before and after the transition to EHR in an academic outpatient ophthalmology practice.

**Methods:**

Review of patient visits per half-day and number of support staff for established faculty ophthalmologists between July and October for five consecutive years beginning the year before EHR implementation.

**Results:**

Eight physicians met inclusion criteria for the study. The number of patient visits was lower in each year after EHR adoption compared to baseline *p* ≤ 0.027). Patient volume per provider was reduced an average of 16.9 % over the 4 years (range 15.3–18.5 %), and during the final year studied, no provider had returned to the pre-EHR number of patients per clinic session. Support staffing was unchanged (*p* > 0.2).

**Conclusions:**

Adoption of EHR was associated with a significantly reduced number of patient visits per clinic session in an academic setting in which support staffing remained stable. Maintaining clinic volume and access in similar settings may require use of additional staffing.

## Background

The adoption of an electronic health record (EHR) represents a major undertaking for those who provide health care in the United States. In 2009, the Health Information Technology for Economic and Clinical Health Act (HITECH) became law, initially giving Medicare and Medicaid providers financial incentives to adopt qualified EHR technology and demonstrate Meaningful Use between 2011 and 2016 but eventually enforcing penalties for non-adopters over time [1–4]. Potential advantages of EHRs over paper charts include electronic exchange of health information, increased patient safety and information security, efficient maintenance and retrieval of patient records, and improved communication between providers and patients [4–6].

During the pre-penalty phase, ophthalmologists have been significantly less likely to adopt EHRs compared to family medicine and general practitioners [7, 8]. A 2013 survey by the American Academy of Ophthalmology revealed 32 % of practices used an EHR; in an additional 15 %, EHRs were partially implemented or was being implemented [9]. In contrast, a 2012 U.S. survey found 72 % of office-based physicians had adopted EHRs [10]. Major barriers to EHR use among ophthalmologists or other providers in ambulatory settings include high initial cost, fewer number of providers per practice, time and training requirements, concern over efficiency and productivity, difficulty with importing old data and with customization such as adding ophthalmic drawings or storing imaging, and alteration of the physician-patient relationship [9, 11–15]. Studies on EHR adoption in ambulatory settings have demonstrated mixed effects on subsequent patient volumes [16–18].

Few studies have evaluated the effects of EHR adoption in ophthalmology. The purpose of this study was to examine the effect of EHR adoption on outpatient clinic volume for four consecutive years following implementation in an academic ophthalmology department.

## Methods

Our study was exempt from Institutional Review Board approval. Data were collected from physicians with established practices at the University of Washington Department of Ophthalmology, including subspecialists in all major ophthalmic specialties. All faculty practices were eligible for inclusion in the study. This study was conducted as a one-group pretest-posttest observational design according to Campbell’s *Experimental and Quasi-Experimental Designs for Research* [19]. Providers with an established practice, defined as at least 3 years of practice at our institution’s ophthalmology clinic at the time of baseline data collection, were included. Due to the logistical challenges of data collection, we chose to study a representative 4-month sample period rather than analyze data from 12-month intervals. The same 4-month period (July through October) was evaluated for 2008–12; the EHR system was instituted in the ophthalmology clinic on May 5, 2009.

After adoption of EpicCare Ambulatory with ophthalmology suite (Epic Systems, Verona, WI), physician templates were intentionally reduced by 50 % for 2 weeks and then by 25 % for 2 weeks, after which time the pre-EHR template was reinstated, although providers were not forced to increase their patient load to that level. Paper charts were available for reference for the first 2 months. Scanned paper records up to 26 months prior were also available in a separate EHR for review throughout the study period. Full-time information technology support staff was present in clinic for 3 months and were available by telephone as needed afterward.

Clinic reports that detailed staffing and the number of completed patient appointments were retrospectively reviewed. At our institution, clinics are held in half-day blocks, and this is the standard “unit” of clinic utilization. The actual number of half-day clinics held by each provider was determined, with clinics outside of the primary practice site of the Department and specialty laser clinics excluded. The number of visits per half-day clinic was calculated from the total visits divided by the total number of half-day clinics. The analysis excludes operating room encounters and specifically-labeled procedure clinics (e.g. glaucoma or retinal laser clinics), which were not included in the analysis. Support staff included residents, fellows, nurses, and technicians; all were counted as 1.0 staff. An exception was made for first year residents, who were assigned a value of 0.5, because they were in their first 4 months of ophthalmology training and were not able to evaluate patients as quickly as more senior residents. Duties of staff and physicians remained unchanged after EHR adoption. Electronic prescribing was highly encouraged from the start of EHR use but was not mandatory.

On July 7, 2009, the Department moved to a new location four miles from the original location. Physician templates were reduced by 50 % from the pre-EHR level for two weeks for this transition, but were then returned to pre-EHR template levels. The percentage of missed appointments for each study period was calculated to evaluate if the change in location resulted in higher no-show rates. For comparison, patient volumes during the same time periods as were examined for the Ophthalmology Department were obtained for two physicians in the University of Washington Department of Internal Medicine who moved clinics from the same original location to the same new location in 2010 but who continued using paper charts.

Data were managed using a spreadsheet program (Excel for Mac 2011; Microsoft, Redmond, WA). Statistical analysis was performed with SPSS 19 for Mac (IBM SPSS Statistics, Chicago, IL), using both a paired, two-tailed t-test and general linear model, repeated-measures ANOVA, with application of the Greenhouse-Geisser correction due to non-sphericity of the data. Correlation of physician years of practice with patient volume and change in patient volume during the study was also analyzed.

## Results

Eight physicians, covering almost all ophthalmic subspecialties, met the inclusion criteria. The median number of years of practice for the physicians included was 11; no other physician data were recorded to ensure provider anonymity due to the small number of providers. Table 1 shows the average number of patients seen for the eight physicians studied, and Table 2 shows patient volume for each study period for each physician. Two providers stopped holding clinics at the institution during the course of the study 3 and 4 years after EHR implementation, respectively, though both continued as faculty in the Department at the Veterans Administration Puget Sound Health Care System. In neither case did the decision to change practice affiliation, made in the second half of the academic year (January-June), affect the physician template or scheduling during the study because the time period evaluated was well in advance of the decision to change positions.

**Table 1:** Patient visits and support staff per provider (N) per half-day clinic session, by year

	2008 (*N* = 8)	2009 (*N* = 8)	2010 (*N* = 8)	2011 (*N* = 7)	2012 (*N* = 6)
Patients per clinic session (range)	14.9 ± 4.0 (9.6-20.1)	12.0 ± 3.2 (7.8-15.5)	11.9 ± 3.1 (7.7-15.0)	12.6 ± 3.0^a^ (8.5-16.5)	12.1 ± 4.2^b^ (6.8-16.7)
Change from 2008 (%)	—	−17.0 ± 6.1	−16.9 ± 12.0	−15.3 ± 10.1	−18.5 ± 6.7
p value*	—	0.002	0.001	0.003	0.027
p value**	—	0.001	0.005	0.014	< 0.001
Support staff per clinic session (range)	2.8 ± 0.6 (2.0-3.5)	2.6 ± 0.3 (2.0-3.1)	2.5 ± 0.3 (2.1-2.9)	2.7 ± 0.6^a^ (2.0-3.4)	3.1 ± 0.6^b^ (2.4-4.1)
Change from 2008 (%)	—	−7.1	−10.7	−3.6	+10.7
p value*	—	0.379	0.248	0.516	0.204
p value**	—	0.379	0.204	0.659	0.573

*N* = number of providers

*Repeated measures ANOVA, Greenhouse-Geisser correction

**Paired, two-tail t test

^a^For 2011, the 2008 baseline = 15.4 ± 4.1 patients and 2.8 ± 0.6 support staff per clinic session for the 7 providers

^b^For 2012, the 2008 baseline = 15.3 ± 4.5 patients and 2.9 ± 0.5 support staff per clinic session for the 6 providers

**Table 2:** Average number of patients seen per half day clinic for each faculty physician included in this study

Faculty	2008	2009	2010	2011	2012
1	20.1	14.7	14.1	14.0	16.6
2	19.5	15.5	15.0	15.7	16.7
3	17.4	14.2	14.8	16.5	13.1
4	15.7	14.8	11.4	10.7	—
5	14.6	11.2	14.7	12.6	11.3
6	11.6	8.1	7.9	—	—
7	10.7	9.6	9.8	9.9	7.9
8	9.6	7.8	7.7	8.5	6.8

For each year after EHR implementation, fewer patients were seen per clinic than in the baseline year (4-year average of 16.9 %, *p* ≤ 0.027 with ANOVA; *p* ≤ 0.014 with paired t-test) (Tables 1 and 2). The average number of support staff for each provider and the number of patients seen per support staff were not significantly different over the same time period (*p* > 0.2). The mean reduction in patient volume was 17.0 ± 6.1 %, 16.9 ± 12.0 %, 15.3 ± 10.1 %, and 18.5 ± 6.7 % at 1, 2, 3, and 4 years after EHR adoption. Up to 4 years after EHR adoption, no physician saw the same number of patients as prior to EHR adoption, with the exception of one time period for one physician (Table 2).

The number of years that each physician had been in practice was not correlated with the baseline number of patients seen (Pearson R = -0.317, *p* = 0.445), patient volume (R range -0.401 to -0.062, *p* ≥ 0.371), or change in patient volume (R range -0.502 to +0.461, *p* ≥ 0.226).

No-show rates obtained for each year remained relatively constant over the period studied (2008 baseline 6.4 ± 0.4 %, range 6.3 ± 0.4 to 7.8 ± 1.0 over the next 4 years; *p* = 0.091) despite the change in practice location in 2009. For the two providers from the Department of Internal Medicine who moved a portion of their practice from the same prior location to the same new location as the ophthalmology practice in 2010 but kept paper charts, patient volume increased each year after the move compared to the baseline year (2009 baseline = 567 patients, range 578 to 773 patients over the next 4 years). Staffing levels for those physicians was unchanged (data not shown).

## Discussion

In the setting of an academic outpatient ophthalmology practice, we found consistent, significant decreases in patient volume that persisted even 4 years following EHR adoption. Our study is limited in scope due to our sample size; still, this decrease is noteworthy and worrisome. Combined with an aging population and a growing need for ophthalmology services [20], a decrease in provider capacity with EHR use may mean that projected ophthalmology workforce shortages [20] are actually being underestimated. Increases in staff, including the use of scribes to enter data and write chart notes [21, 22], have been shown to improve patient flow in other specialties but represent an additional, unforeseen expense of EHR adoption.

Our study has several limitations. We examined results from a small number of providers in a single academic practice. Personnel changes over time resulted in a smaller pool of data in the later years of the study, though our study findings were significant and very consistent across all included physicians despite the small sample size. The change in location theoretically could have impacted the numbers of patients seen, but this appears unlikely in this case. The providers all had established practices, and no-show rates remained essentially stable throughout the years studied. The move would not be expected to continue to affect patient volumes 3 or 4 years later. Furthermore, we analyzed internists who made the same change in location while using paper charts and actually experienced an increase in patient volume, without change in staffing levels. We selected a 4-month study period for our experimental and control group to limit the painstaking data collection process of manually matching exact physician clinic dates, patient appointments, and support staff as well as to limit the effect of any changes in physician employment that occur in July of each year. For example, if a faculty member left the Department in July, patient volumes during January through June could have been affected. Additionally, we wanted to avoid seasonal variation (particularly holidays in November and December) as well as avoiding the Spring months prior to EHR adoption as the data may be skewed by provider desire to overbook prior to implementation. The patient volume increased in our control group while decreased in our study group over the 4-month period for 4 consecutive years.

During our study period, no major changes in the clinic’s policy, operations, or clinical practice standards occurred that could have explained the findings. The clinic session length was unchanged and the number of staff per clinic was similar and not significantly different during the study period. No changes to workflow were made on an organizational level during our study but individual changes to workflow could have occurred. Physicians were able to reduce their templates from pre-EHR levels at any time, so it is unclear if they could have seen the same volume of patients if forced to. However, external factors (patient access, facility staffing based on volume expectations, and a physician pay incentive plan based on productivity) strongly encouraged the faculty to maximize patient throughput. Individual template reductions would not be expected to impact patient volumes across all providers and would not likely persist for 4 years given the aforementioned incentives to increase patient volume.

With the exception of an “optimization event” in December 2011 consisting of time and motion studies undertaken for every physician in the Department aimed at improving provider facility with the EHR, no other quality improvement measures were implemented. This optimization event found the average time per patient visit was approximately 19 min from entering the exam room to closing the patient chart, and more than 90 % of physicians closed charts after completion of their clinic. Pre-EHR data are not available for comparison, but this initiative did not increase the average number of patients seen per clinic session in the subsequent year included in our study, suggesting that senior physicians perceived to be less facile with EHR were not associated with the decline in patient volume. Although we did not measure time spent performing specific tasks during each encounter, we conjecture that the added time spent in the exam room could be due to charting rather than face-to-face interaction and examination, and is supported by other authors’ findings [16, 23, 24].

Finally, the EHR interface itself was not unreasonably cumbersome to use, though no subjective data are available comparing its ease of use amongst the providers, nor comparing it to other EHR systems. The impact of any software updates and/or interface customizations during the time of the study on the user experience is similarly unknown. Like many large academic institutions, the criteria for adoption of a specific EHR system were largely based upon how well the EHR fit the entire enterprise across numerous medical specialties, and our small department had no direct input into the EHR vendor chosen; this lack of user involvement represents one key pitfall in EHR adoption change management.. Lastly, documentation requirements within the EHR system met the minimum “meaningful use” criteria and each provider documented in the chart to the level of their satisfaction. Informal review of providers’ charts did not reveal systematic over-documentation, though it is possible that such action could affect time spent in charting considerably. The department did not use scribes or other personnel to assist with documentation before and after EHR adoption.

Other studies in academic ophthalmology clinics have found mixed effects from EHR adoption [23–27]. A large study of an entire department reported increased provider non-clinic documentation time with EHR, 6.8 min more per encounter versus paper charting, but with only a 3 % decrease in clinic volume at 2 and 3 years after implementation. However, no mention is made of clinic staffing levels during the study period [23]. A subset of 4 pediatric ophthalmology providers at that institution had an 11 % decrease in clinical volume at 3 years following EHR implementation, although this was not statistically significant [25].

Other studies have reported no significant change in patient volume. One glaucoma subspecialty practice reported that more time was spent with patients by faculty and fellows in the exam room (both directly talking to or examining the patient as well as talking to the patient while on the computer) compared to baseline. No additional staff or clinic space was added, but the patient volumes for the year pre-EHR adoption and the year post-EHR were unaffected; this was felt to be due to documentation being performed during the patient encounter, rather than in-between patient encounters as occurred pre-EHR [24]. Another academic ophthalmology department reported no significant change in patient volume per provider 4 years after adoption of EHR when compared to the 5 years prior to EHR, for 12 ophthalmologists and optometrists with established practices (at least 3 years) prior to the study [26]. However, changes in staffing were not reported, and it is unclear if all providers maintained the same clinical load during the study.

Another study of an academic department consisting of 23 physicians reported no significant change in total patient volume, although glaucoma specialists significantly increased their volume [27]. However, the authors noted that the department had access to substantial financial and human resources, including a large capital budget for optimization and support staff that was temporarily redistributed during the implementation process. Additionally, their ophthalmology department was the last of the hospital departments to implement EHR and learned from other transitions. In contrast, our department was the first non-primary care specialty to adopt EHR in our hospital system, though no improvement occurred after an optimization event held 2 years after EHR adoption. However, the event was limited to a single department and there are no data on how many other departments transitioned during the interval time period.

Data on EHR implementation and effects on other specialties and practices are also limited. One study from an academic primary care pediatric clinic reported a 10 % decrease in patient volume for 3 months post-EHR and increased length of clinic visit time compared to baseline 2 years later [16]. A study from a large academic multispecialty group found small increases in ambulatory patient volume 6 months after EHR adoption, but the authors felt that some visit types that were previously undocumented (such as lab visits, vaccinations, or counseling visits) were now being counted [17].

Studies of EHR implementation are inherently difficult to compare because of differences in software, staffing, and practice characteristics. Furthermore, many physician behaviors that affect patient volume and time per patient are difficult to capture. For instance, some authors have found that physicians spend substantial amounts of time charting outside of regular business hours in practices with EHR [23, 25]. A provider who documented after hours might then be able to see more patients with EHR but end up spending significantly more time to do so. In addition, electronic prescribing can take significantly more time than writing prescriptions [28], and this was implemented simultaneously with EHR at our institution.

Importantly, our study provides additional data on EHR implementation and its potential effects on ophthalmologists given the scarcity of literature on this subject. Although our study has its limitations, our results indicate that EHR implementation may lead to persistent decreases in patient volume. Our study does not investigate why the decreases in volume occurred; rather we are simply reporting the data from our Department’s transition to EHRs. Our study did not measure time spent examining patients compared with charting, nor did it measure quality of care, patient or provider satisfaction, or outcomes in other specialties within the institution. Further studies in a variety of practice settings are necessary to provide guidance on the impact of EHR adoption on ophthalmology in the United States.

## Conclusions

Adoption of EHRs in our academic ophthalmology practice with no change in support staffing was significantly associated with reduction in patient volume. Additional staffing may be necessary to maintain clinic volume and access to care in similar settings. Decreases in provider capacity due to EHR use may represent a new barrier to accessing ophthalmic care in the United States in combination with a growing need for ophthalmology services and projected physician workforce shortages. Better user involvement and change management practices may also be helpful in guiding future EHR adoptions. Future quantitative and qualitative studies (focus groups, in-depth interviews, or ethnography) can provide further data on the effect of EHRs on ophthalmology practices and examine more closely the impact of EHRs on the time actually spent in the exam room with patients compared with charting.

## Abbreviations

EHR: electronic health record
HIT: Health Information Technology
HITECH: Health Information Technology for Economic and Clinical Health

## References

[1] The Health Information Technology for Economic and Clinical Health (HITECH) Act, ARRA Division A Title XIII and Division B Title IV. 2009. http://www.gpo.gov/fdsys/pkg/BILLS-111hr1enr/pdf/BILLS-111hr1enr.pdf. Accessed 29 May 2015.

[2] Centers for Medicare and Medicaid Services. EHR Incentive Program. http://www.cms.gov/Regulations-and-Guidance/Legislation/EHRIncentivePrograms/index.html?redirect=/EHRIncentiveprograms. 2015. Accessed 29 May 2015.

[3] Centers for Medicare and Medicaid Services. Payment Adjustments and Hardship Tipsheet for Eligible Professionals. http://www.cms.gov/Regulations-and-Guidance/Legislation/EHRIncentivePrograms/Downloads/PaymentAdj_HardshipExcepTipSheetforEP.pdf. 2014. Accessed 29 May 2015.

[4] Boland MV (2010). Meaningful use of electronic health records in ophthalmology. Ophthalmology.

[5] Nguyen L, Bellucci E, Nguyen LT (2014). Electronic health records implementation: an evaluation of information system impact and contingency factors. Int J Med Inform.

[6] Mertz L (2012). Electronic health records usher in a new era in health care. IEEE Pulse.

[7] Grinspan ZM, Banerjee S, Kaushal R, Kern LM (2013). Physician specialty and variations in adoption of electronic health records. Appl Clin Inform.

[8] Kokkonen EW, Davis SA, Lin HC, Dabade TS, Feldman SR, Fleischer AB (2013). Use of electronic medical records differs by specialty and office settings. J Am Med Inform Assoc.

[9] Boland MV, Chiang MF, Lim MC, Wedemeyer L, Epley KD, McCannel CA (2013). Adoption of electronic health records and preparation for demonstrating meaningful use: an American Academy of Ophthalmology survey. Ophthalmology.

[10] Hsiao CJ, Hing E (2012). Use and characteristics of electronic health record systems among office-based physician practices: United States, 2001-2012. NCHS Data Brief.

[11] DeBry PW (2001). Considerations for choosing an electronic medical record for an ophthalmology practice. Arch Ophthalmol.

[12] Chiang MF, Boland MV, Brewer A, Epley KD, Horton MB, Lim MC (2011). Special requirements of electronic health record systems in ophthalmology. Ophthalmology.

[13] Miller RH, Sim I (2004). Physicians’ use of electronic medical records: barriers and solutions. Health Aff (Millwood).

[14] Ayatollahi H, Mirani N, Haghani H (2014). Electronic health records: what are the most important barriers?. Perspect Health Inf Manag.

[15] Kruse CS, Regier V, Rheinboldt KT (2014). Barriers over time to full implementation of health information exchange in the United States. JMIR Med Inform.

[16] Samaan ZM, Klein MD, Mansour ME, DeWitt TG (2009). The impact of electronic health record on an academic pediatric primary care center. J Ambul Care Manage.

[17] Cheriff AD, Kapur AG, Qiu M, Cole CL (2010). Physician productivity and the ambulatory EHR in a large academic multi-specialty physician group. Int J Med Inform.

[18] Swanson T, Dostal J, Eichhorst B, Jernigan C, Knox M, Roper K (1997). Recent implementations of electronic medical records in four family practice residency programs. Acad Med.

[19] Campbell DT, Stanley JC (1963). Experimental and quasi-experimental designs for research.

[20] U.S. Department of Health and Human Services. Physician Supply and Demand: Projections to 2020. http://bhpr.hrsa.gov/healthworkforce/supplydemand/medicine/physician2020projections.pdf. 2006. Accessed 29 May 2015.

[21] Arya R, Salovich DM, Ohman-Strickland P, Merlin MA (2010). Impact of scribes on performance indicators in the emergency department. Acad Emerg Med.

[22] Bank AJ, Obetz C, Konrardy A, Khan A, Pillai KM, McKinley BJ (2013). Impact of scribes on patient interaction, productivity, and revenue in a cardiology clinic: a prospective study. Clinicoecon Outcomes Res.

[23] Chiang MF, Read-Brown S, Tu DC, Choi D, Sanders DS, Hwang TS (2013). Evaluation of electronic health record implementation in ophthalmology at an academic medical center (an American Ophthalmological Society thesis). Trans Am Ophthalmol Soc.

[24] Pandit RR, Boland MV (2013). The impact of an electronic health record transition on a glaucoma subspecialty practice. Ophthalmology.

[25] Redd TK, Read-Brown S, Choi D, Yackel TR, Tu DC, Chiang MF (2014). Electronic health record impact on productivity and efficiency in an academic pediatric ophthalmology practice. J AAPOS.

[26] Lim MC, Patel RP, Lee VS, Weeks PD, Barber MK, Watnik MR (2015). The longer-term financial and clinical impact of an electronic health record on an academic ophthalmology practice. J Ophthalmol.

[27] Singh RP, Bedi R, Li A, Kulkami S, Rodstrom T, Altus G (2015). The practice of electronic health record system implementation within a large multispecialty ophthalmic practice. JAMA Ophthalmol.

[28] Devine EB, Hollingworth W, Hansen RN, Lawless NM, Wilson-Norton JL, Martin DP (2010). Electronic prescribing at the point of care: a time-motion study in the primary care setting. Health Serv Res.

